# Citizen Science as a New Tool in Dog Cognition Research

**DOI:** 10.1371/journal.pone.0135176

**Published:** 2015-09-16

**Authors:** Laughlin Stewart, Evan L. MacLean, David Ivy, Vanessa Woods, Eliot Cohen, Kerri Rodriguez, Matthew McIntyre, Sayan Mukherjee, Josep Call, Juliane Kaminski, Ádám Miklósi, Richard W. Wrangham, Brian Hare

**Affiliations:** 1 Canine Research Department, Canines, Inc., Durham, NC, United States of America; 2 Department of Evolutionary Anthropology, Duke University, Durham, NC, United States of America; 3 School of Anthropology, University of Arizona, Tucson, AZ, United States of America; 4 Statistics Department, 23 and me, Inc., Mountain View, CA, United States of America; 5 Departments of Statistical Science, Computer Science, Mathematics, Duke University, Duke University, Durham, NC, United States of America; 6 Department of Psychology and Neuroscience, University of St Andrews, St Andrews, United Kingdom; 7 Department of Psychology, Max Planck Institute for Evolutionary Anthropology, Leipzig, Germany; 8 Department of Psychology, University of Portsmouth, Portsmouth, United Kingdom; 9 MTA-ELTE Comparative Ethology Research Group, Budapest, Hungary; 10 Department of Human Evolutionary Biology, Harvard University, Cambridge, MA, United States of America; 11 Center for Cognitive Neuroscience, Duke University, Durham, NC, United States of America; Università di Parma, ITALY

## Abstract

Family dogs and dog owners offer a potentially powerful way to conduct citizen science to answer questions about animal behavior that are difficult to answer with more conventional approaches. Here we evaluate the quality of the first data on dog cognition collected by citizen scientists using the *Dognition*.*com* website. We conducted analyses to understand if data generated by over 500 citizen scientists replicates internally and in comparison to previously published findings. Half of participants participated for free while the other half paid for access. The website provided each participant a temperament questionnaire and instructions on how to conduct a series of ten cognitive tests. Participation required internet access, a dog and some common household items. Participants could record their responses on any PC, tablet or smartphone from anywhere in the world and data were retained on servers. Results from citizen scientists and their dogs replicated a number of previously described phenomena from conventional lab-based research. There was little evidence that citizen scientists manipulated their results. To illustrate the potential uses of relatively large samples of citizen science data, we then used factor analysis to examine individual differences across the cognitive tasks. The data were best explained by multiple factors in support of the hypothesis that nonhumans, including dogs, can evolve multiple cognitive domains that vary independently. This analysis suggests that in the future, citizen scientists will generate useful datasets that test hypotheses and answer questions as a complement to conventional laboratory techniques used to study dog psychology.

## Introduction

Dogs have become a favorite species of comparative psychologists over the last decade[1, 2]. This recent research has revealed a number of ways dogs seem to solve social problems more similarly to human infants than many primates[3, 4]. Comparisons to other species suggest the possibility that some of these skills are the result of domestication [2, 3, 5–9] but see [10, 11]. A variety of laboratory studies have begun to map out in detail cases where dogs show cognitive flexibility and cases where they are constrained or show biases in their problem solving [12–20]. Interest in studying dogs across disciplines is increasing because of their ubiquity, the low cost of non-invasive research on family dogs, and the potential applications of research for working dogs [21].

Dog cognition researchers have modeled their approach after developmental psychologists studying human infants. Dog owners are invited to bring their family dogs into a space provided by a university to observe the dog’s behavior or researchers travel to a family’s home or “doggy daycares” to study dogs where they are cared for [1, 2]. While emulating the approach of developmental psychologists has been profoundly productive, it is not without limitations. Similarly to developmental psychology, the majority of research involves testing 10–50 individuals to make species-level conclusions about a single cognitive skill (but for exceptions see (sample size: 5, sample size and measures: [22, 23])). The current techniques to study dog cognition may have only begun to reveal the true potential of research with dogs. With tens of millions of family dogs worldwide, if it became feasible to collect large quantities of quality cognitive data, the study of within-species individual differences could be revolutionized.

A major prediction of the cognitive approach is that cognitive architecture differs among individuals and species because multiple domains of cognition exist and can vary independently [24, 25]. The underlying assumption is that selection can act on these different types of cognitive abilities to produce within- and between-species variability in cognitive profiles [6, 24, 26]. This stands in direct opposition to any theoretical stance suggesting animal cognition can be described by a uni-dimensional measure of intelligence (e.g. learning ability, etc. [25]). The strongest within-species test of this hypothesis with nonhumans assessed over a hundred chimpanzees on the same battery of cognitive tests. Two cognitive factors best explained individual differences in chimpanzee performance—a general cognitive factor and spatial memory [27]. No non-primate species has been tested in a similar way. Dogs are an ideal species in which to investigate the structure of non-primate cognition, since it is possible to recruit large samples. However, the ideal sample size of hundreds of dogs is beyond what conventional laboratory approaches have typically been able to achieve.

Citizen science has been used by a variety of biologists requiring seemingly overwhelming amounts of data [28–30]. Citizen science can vary in its exact definition and how it is implemented, but most projects use unpaid online volunteers (“crowdsourcing”) to collect large-scale data [31]. Recently citizen science has seen rapid growth across a range of disciplines including evolution [32], ecology [33], astronomy [34] and conservation research [35]. In order to enhance our ability to answer questions about dog cognition we created the citizen science website *Dognition*.*com*. Building on past success using owner surveys [36, 37] *Dognition*.*com* shows dog owners how to conduct a series ten simple cognitive experiments. Anyone in the world with Internet, a dog, and a few basic household items (i.e. paper, post-it notes, cups, and dog treats) can pay to join and participate. Using large datasets produced through crowdsourcing we can then begin to examine questions that otherwise might not be tractable—such as examining individual differences in dog cognition.

While the use of crowdsourcing is well established, no previous citizen science project has involved non-scientists conducting behavioral experiments. The study of animal cognition has a long tradition of developing techniques to reduce experimenter bias. For example, it is standard practice to videotape laboratory psychology experiments so that subtle behaviors that cannot be coded live can be evaluated and reliability assessments can be conducted [38, 39]. Without this check there is always the real concern that even the best-trained experimenters might unconsciously influence subjects’ choices through cuing or by making other important procedural errors that might go undetected (although see [40, 41]). Thus, it is reasonable to question whether dog owners can follow instructions well enough to conduct behavioral experiments and produce scientifically useful data.

In this paper we 1) provide an initial evaluation of the quality of data produced by citizen scientists using Dognition.com and 2) examine whether the performance of the first 522 dogs tested is best explained by either a single or multiple cognitive factors.

## Methods

Once a dog owner joins the Dognition.com service and provides demographic information about their dog, s/he is provided with a questionnaire as well as written and video instructions on how to carry out ten cognitive exercises. Both the questionnaire that is largely derived from Hsu & Serpell [36] and the cognitive exercises are based on previously published research. Participant responses are input through a standardized interface across digital platforms (i.e. PCs, smart phones, tablets, etc.). The interface provides clear choices to participants about their dogs’ past behavior or decisions during the cognitive exercises (e.g. did your dog choose the food on your left or right?). All data is submitted to *Dognition*.*com* and stored on servers for the purpose of providing dog owners’ with a report on their dog and for testing hypotheses regarding dog cognition. To encourage participants to collect meaningful data, exercises are not framed as tests that are either passed or failed, but rather as measures to reveal the strategies their dog relies on relative to other dogs tested in the same exercises. No information is provided that suggests what hypotheses might be tested with group data.

### Participants

All exercises were first piloted with thirty volunteers and their dogs while members of our team observed. In this pilot phase, exercises were retained in the battery and instructions modified based on the ability of participants to carry out tests correctly and maintain their dogs’ motivation. This means methodological differences exist between previously published versions of each study and the version carried out by participants here. A Beta version of the service was then created and advertised via dog-related email listings. Only those replying to the recruitment emails could sign up and participate during this free trial period prior to the public launch of Dognition.com. This Beta dataset includes 245 participants and their dogs that completed all the tasks provided. Dognition.com was launched publicly on February 5, 2013 with participation during the initial period requiring a membership costing ~$60. Participants could learn about participating from media reports, emails, or other online sources (blogs, Facebook, etc.). The “Live dataset”, collected after the public launch, is made up of our first 277 dogs. Thus almost half of our 522 citizen scientists participated for free (46.9%) while a little over half paid for access (53.1%). The age and sex breakdown of the dogs tested by each group were relatively balanced (Table 1). During the Live period, participants had the opportunity to register through a page designed for professional dog trainers. The use of third party data from Dognition.com was approved by Duke University IACUC protocol A138-11-06.

**Table 1:** Age and sex of subjects in Beta, Live and combined datasets.

	Females	Males	Total
**Beta**			
0 to 1 years	17	24	41
2 to 6 years	76	63	139
7 or more years	33	32	65
**Total**	126	119	245
**Live**			
0 to 1 years	22	23	45
2 to 6 years	82	90	172
7 or more years	24	36	60
**Total**	128	149	277
**Combined**			
0 to 1 years	39	47	86
2 to 6 years	158	153	311
7 or more years	57	68	125
**Total**	254	268	522

### Procedure

Table 2 provides general descriptions of each exercise while detailed methods are provided in the supplemental information. All participants received instructions recommending they play the cognitive exercises at home in a familiar room where their dog was comfortable (e.g. familiar and water available *ad libitum*) and reasonably free of distractions. It is also suggested that breaks be taken whenever the dog lost motivation (e.g. in retrieving treats, etc.). Participants were repeatedly reminded implicitly and explicitly not to intentionally influence their dog’s choices, although experimental evidence suggests that the effect of unconscious cuing on dogs during cognitive experiments may not be as ubiquitous as has often been suggested [42]. Schmidjell et al [41] and Hegedüs et al [40] both varied owners’ knowledge about where a reward was hidden as well as their belief about whether their dog was expected to follow a human point to be correct. No evidence was observed for owners influencing dogs in these cognitive tasks even when the experiment was designed to encourage it (also see [11, 42]). Regardless, wording was placed at registration and throughout the instructions to remind participants that there “are no correct answers” and to let their dog choose freely.

**Table 2:** The numbers of trials, the order tasks are presented to all participants, task names and general methods. For more details see supplemental methods.

# of Trials	Order	Task	General Method
1	1	Yawn control	Participant says “yellow” every 5s for 30s.
1	2	Yawn Exp.	Participant yawns every 5s for 30s.
3	3	Eye Contact Warm Up	Participant holds food to her face for 10s and gives treat to dog.
3	4	Eye Contact	Participant holds food to their face and record when and if dog breaks eye contact during 90s countdown.
6	5	Treat Warm Up	Participant introduces the two locations to find food and allows dog to retrieve.
6	6	Arm Pointing	Participant extends her arm and index finger toward one of two food pieces placed on floor and allows dog to retrieve.
6	7	Foot Pointing	Participant extends their leg and foot toward one of two food pieces placed on floor and allows dog to retrieve.
2	8,11	Watching	Participants face their dog, verbally forbid them from taking food and record when and if they retrieve food during 90s countdown.
2	9	Back Turned	Same as Watching except participants turn their back after placing food.
2	10	Eyes Covered	Same as Watching except participants cover their eyes with their hands after placing food.
4	12, 13	1 & 2-Cup Warm Up	a) Participant shows dog food being hidden under one cup b) Participant place two cups and shows dog food being hidden in one of two cups.
6	14	Memory vs. Pointing	Same as 2-cup warm up plus participant points to empty cup.
4	15	Memory vs. Smell	Same as 2-cup warm up plus participant occludes dog view as treat is moved to empty cup.
4	16	Delayed Memory	Same as 2-cup warm up with increasing delays each trial (60, 90, 120 and 180 seconds) until dog is released to search.
6	17	Inferential Reasoning Warm Up	Participant hides food in one of two cups. Picks up cups showing dog which cup contains food. Places cups back in position. Picks up empty cup, puts it back and releases dog.
4	18	Inferential Reasoning Task	Same as Warm Up except dog is not shown in which cup food is hidden.
4	19	Physical Reasoning Warm Up	Participant props a piece of paper up with a food treat and allows dog to retrieve.
4	20	Physical Causality Task	Same as Warm Up plus 2nd piece of paper placed flat on the ground.

For each task participants were provided with a how-to video that detailed the experimental setup, procedural protocol, and the set of possible responses from their dog that they would need to live code. Written instructions were also given that repeated the steps seen in the video. A Frequently Asked Questions (FAQ) section was provided and was co-located on the same page as the video and written instructions. A link to the FAQ was available at all times to participants in case questions arose during testing.

Participants were provided with each step, one at a time. Participants were not able to advance through tasks or trials out of order. This was designed to make it as easy as possible for participants to correctly follow all steps and repeat each task the correct number of times. Participants advanced through each predetermined step by clicking “next”. Once all of the steps were complete for each trial, participants were asked to code their dog’s behavior live. The majority of decisions involved either stopping a timer or indicating if the dog chose the location with a treat. In every instance that the citizen scientist was prompted to record the dog’s response, they were explicitly reminded, “There are no correct answers” and to enter data accurately. All behavioral codes were recorded by the system immediately upon entry. All participants received the different cognitive exercises in the order shown in Table 2.

**Fig 1:**
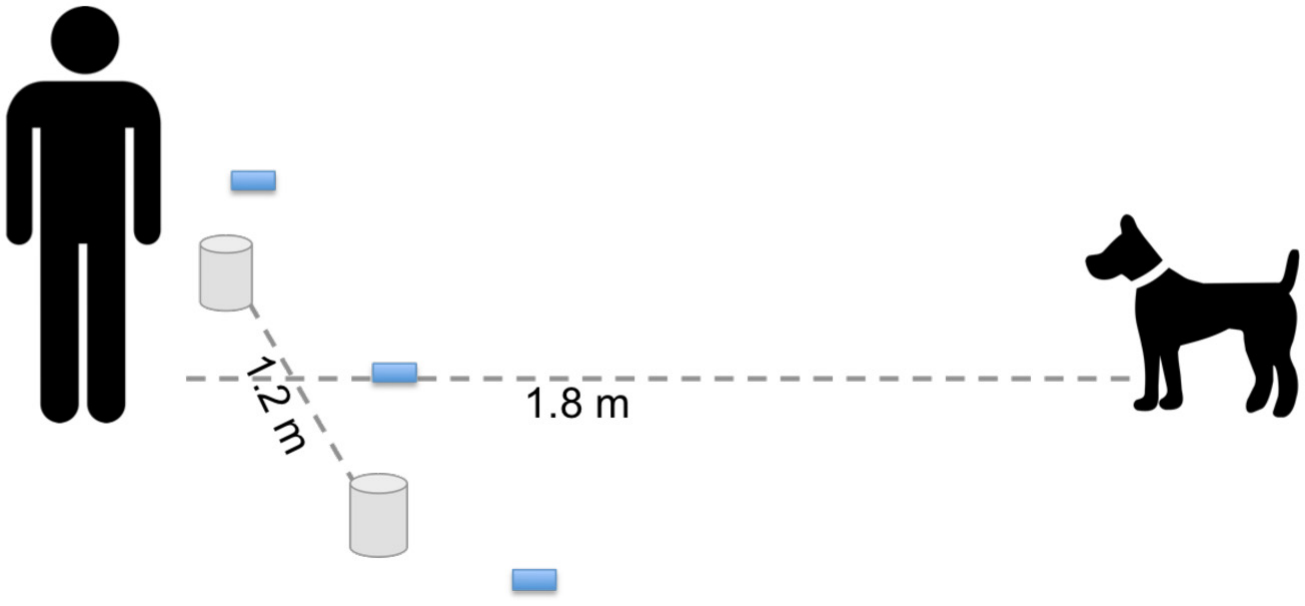
Experimental set up for all experiments requiring dogs to make a choice between one of two hiding locations. Participants were instructed to place treats or cups (represented by cylinders) 1.2 meters apart while standing 1.8 meters from their dog. Three post-it notes (represented by grey squares) were placed on the ground to aid in live coding of a dog’s choice.

Fig 1 shows the experimental setup for the majority of tasks that are two-way choices. Participants were instructed to work with a partner unless their dog was able to sit and stay on their own. In the instructional video the dog was positioned directly across from E (the experimenter), approximately 1.8 meters away. Three Post-it notes were placed on a line perpendicular to the dog and E. The center Post-it was placed .6 meters directly in front of E while the other two were placed .9 meters on either side of the center Post-it.

Once they had prepared the exercise in this way, participants could follow the step-by-step instructions for each trial. For the general procedure of each task see Table 2. After the participants had completed the experimental manipulation for each exercise, they were instructed to release the dog to choose in choice exercises. Either a dog could be released from a sit-stay or their partner released the dog. Video and written instructions emphasized the importance of staring directly ahead during a dog’s choice and for a partner to release the dog without nudging, pushing or leading the dog in one direction or the other. After each choice the system automatically advanced to the next trial and indicated how many times each trial type needed to be repeated for each task.

Non-differential rewarding in which any choice was rewarded was used in arm pointing, foot pointing, eyes covered, back turned, and watching tasks while differential rewarding was used in memory versus pointing, memory versus smell, delayed memory and both reasoning tasks. In all choice tasks where dogs had to locate treats on their first choice in one of two locations the online instructions indicated which side to place the treat for each trial. Counterbalancing was automated so that half of subjects received their first trial on the left or right, while within a test subjects received equal number of trials on the left and right with no location baited more than twice consecutively. For the Live data set a redo button was introduced to actively prompt a participant to repeat a trial in case they felt they made an error. After users submitted their dog’s choice on each trial they could either confirm the choice or use the redo button to try the trial again.

### Coding and Analysis

Video and written instructions demonstrated to participants how to live-code the dependent measure in each exercise. See S1 Table for questions that participants responded to on each trial based on their dog’s choice within the trial. In the two-way choice tasks, a choice was coded when the dog crossed (i.e. with paw or nose) between the plane created between the center-left or center-right Post it notes (initial piloting indicated that post-it notes helped participants make the most accurate judgments; see Fig 1). In the “cunning” conditions participants simply stopped a countdown timer when and if their dog ate the forbidden food. Clear examples of dog yawns and loss of eye contact were shown on video for the yawning and eye contact exercises. The yawning instructions explicitly encouraged participants to be conservative in their coding by stating, “if you are unsure whether your dog yawned, do not record a yawn.” In the eye-contact exercise our dependent measure was again made conservative to aid ease of coding. A break of eye contact was only to be registered if the participant silently counted to two when the dog turned its head to look away (i.e. a shift in face direction). More subtle breaks in eye contact that might traditionally be coded from video (e.g. saccades) would give more resolution but would have made live coding by citizen scientists extremely difficult.

To examine group performance in each task in each dataset we used a one-sample t-test for the majority of tasks since they involved a two-way choice. A repeated measures ANOVA was employed to compare whether dogs’ latencies to take forbidden food differed in the three conditions examining a dog’s use of cues about others’ visual perception. The yawning task was analyzed using a McNemar’s test to compare the number of dogs that yawned in the experimental versus the control condition.

We also conducted quantitative comparisons between citizen science and published lab data collected using the most similar experimental protocols to those used here. To maximize power and conservatively guard against a type II error, the Live and Beta datasets were combined for these analyses. The published data were obtained from many of the papers that each of our methodologies was modeled after (Table 3). This included arm pointing, foot pointing, other’s visual cues, memory versus pointing, memory versus smell, delayed choice, and physical reasoning. The first trial of the delayed memory task was compared to unpublished laboratory data with a highly similar method. Qualitative comparisons were carried out for the yawning and inferential reasoning task where published studies exist but original data was not available. We were unable to locate data that would allow us to carry out a systematic comparison of eye contact data.

**Table 3:** Means, Standard Error, degrees of freedom, test statistic and p-value from the quantitative comparisons between laboratory data and citizen science data collected through Dognition.com. Welch independent t-tests were used for all comparisons except Memory vs. smell and Memory vs. pointing for which a Wilcoxon sign rank test (continuity corrected) was used, and Delayed Memory for which a proportions test was used.

Test	Comparison Publication	Lab		Citizen Scientist (N = 522)				
		Mean	SE	Mean	SE	df	statistic	p-value
Arm Pointing	Gácsi et al, 2009 (N = 180)	67.97%	1.18%	66.28%	0.90%	403.39	t = 1.14	p = 0.25
Foot Pointing	Lakatos et al, 2009 (N = 15)	65.83%	5.51%	64.55%	0.95%	14.84	t = -0.23	p = 0.82
Other’s visual cues	Call et al, 2003 (N = 14) Watching	34.92s	8.81s	46.99s	1.55s	10.63	t = 1.34	p = 0.21
	Eyes Closed	25.50s	8.43s	46.06s	1.66s	11.87	t = 2.39	p = 0.03
	Back Turned	24.63s	8.23s	47.54s	1.63s	11.88	t = 2.73	p = 0.02
Memory vs. Pointing	Szetei et al, 2003 (N = 10)	52%	6.11%	68%	1.40%	Wilcoxon Rank Sum	w = 3573.5	p = 0.04
Memory vs. Smell	Szetei et al, 2003 (N = 10)	12%	4.66%	26.20%	1.25%	Wilcoxon Rank Sum	w = 3295.5	p = 0.13
Delayed Memory	MacLean et. al, unpub. (N = 49)	71%	6.52%	81%	1.70%	1	X^2^ = 2.36	p = 0.12
Physical Reasoning	Bräuer et al, 2006 (N = 24)	66.67%	4.60%	62.60%	1.14%	25.94	t = -0.86	p = 0.40

To analyze the effect of participant bias on results, we analyzed the use of the redo functionality in the Live dataset. First, in tasks where enough data existed (i.e. 20 or more individuals using the redo button) we compared the performance of those who used the redo button to those who did not. If participants were using the redo button to intentionally or unintentionally manipulate results, then a difference should be detected. To analyze the impact of training on performance, the results of dogs owned by professional trainers (n = 51) were compared to those of dogs of non-trainers (n = 226) in the live dataset using a Welch independent t-test. If experience handling dogs or the obedience of the dog, impacts cognitive performance then trainer dogs should differ from non-trainer dogs.

To assess the correlational structure underlying individual differences across tasks we conducted exploratory factor analyses. These analyses were conducted for the Beta and Live datasets separately to assess the consistency of the resulting factor structure in independent samples. To determine the number of factors to extract we conducted parallel analyses comparing the eigenvalues from the actual data to a random resampling of the dataset [43]. These analyses suggested a four-factor solution for both datasets. Factor models were fit using the principal factor solution and an orthogonal (Varimax) rotation.

## Results

**Fig 2:**
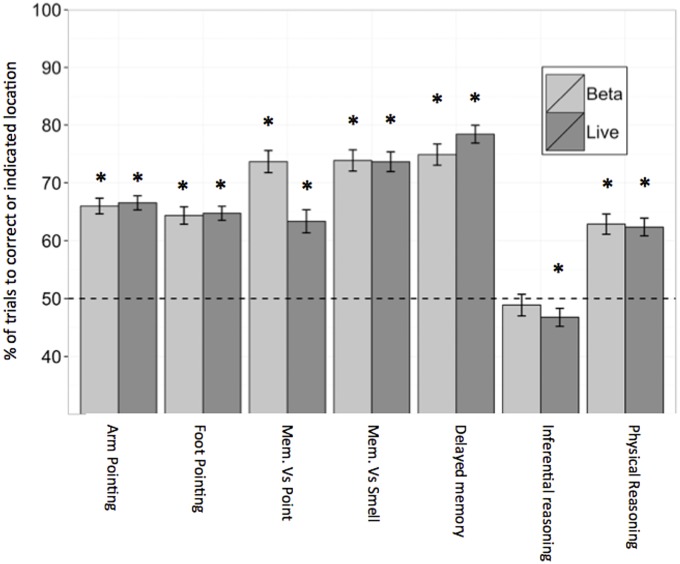
Mean performance (+/- SEM) in each task in which dogs faced a two way choice with chance being 50%. In the memory exercises (Mem Vs. Point and Mem Vs. Smell) success was scored when a dog made a choice consistent with relying on their memory (i.e. not a gesture or olfactory cues). Light grey bars represent Beta and dark grey representing Live datasets. *<0.05 binomial probability.

Table 4 contains all results for each cognitive task in Beta and Live datasets and Fig 2 illustrates success in the two-way choice tasks from the Beta and Live datasets. In the yawning task dogs yawned significantly more in the experimental condition than the control condition in the Beta dataset, whereas the difference between conditions was not significant in the Live dataset. In the arm pointing and foot pointing tasks, dogs in both datasets chose the treat pointed to at a level significantly greater than predicted by chance. In the other’s visual cues task there was no difference in how long dogs waited to retrieve forbidden food in the Beta dataset, however there was a significant difference between conditions in the Live dataset. Pairwise t-tests, with Bonferroni correction were used as post hoc tests on the Live dataset for the 3 conditions of the other’s visual cues task. These tests revealed that dogs ate the prohibited food significantly sooner during the eyes covered condition than in the watching or back turned conditions, but that the back turned and watching conditions did not differ. In the memory versus pointing, memory versus smell, and delayed tasks, dogs chose the location where they had seen the food hidden significantly more than where they were directed by a gesture or where they could have smelled the food. In the physical reasoning task, dogs had a significant preference for choosing the paper propped up by the hidden treat. In the inferential reasoning tasks, dogs tended to choose the location without the food (i.e. the empty cup last touched by a human), however this difference was only significant in the Live dataset.

**Table 4:** Descriptive and test statistics comparing experimental (E) and control conditions (C) in each task of the Beta (N = 245) and Live (N = 277) datasets. All tests were One-Sample T-tests except visual cues tasks (Repeated ANOVA) and Yawning (McNemar’s).

			Beta Data					Live Data			
Exercise	Trials	Mean	SE	Stat	DF	p	Mean	SE	Stat	DF	p
Yawning				ChiSq = 5.513	1	0.018			Chisq = 0	1	1
Control	1	C: 44/245					C: 65/277				
Experimental	1	E: 66/245					E: 64/277				
Eye Contact	3	40.46 s	1.5	NA		NA	46.38 s	1.41	NA		NA
Arm Pointing	6	3.96	0.08	11.851	244	**<.001**	3.99	0.07	13.557	276	**<.001**
Foot Pointing	6	3.86	0.09	9.519	244	**<.001**	3.88	0.07	12.192	276	**<.001**
Other’s Visual Cues				F = .733	2,488	0.481			F = 6.115	2,552	**0.002**
Watching Condition	2	42.57s	2.24				50.92s	2.12			
BackTurnedCondition	2	42.79s	2.43				51.75s	2.18			
EyesCoveredCondition	2	44.18s	2.47				47.74s	2.23			
Memory vs. Pointing	6	4.42	0.12	12.342	244	**<.001**	3.8	0.12	6.7	276	**<.001**
Memory vs. Smell	4	1.05	0.07	12.884	244	**<.001**	1.05	0.07	13.819	276	**<.001**
Delayed Memory	4	3	0.07	13.591	244	**<.001**	3.14	0.06	18.567	276	**<.001**
Inference Reasoning	4	1.96	0.08	0.597	244	0.551	1.87	0.06	2.084	276	**0.038**
Physical Reasoning	4	2.51	0.07	7.394	244	**<.001**	2.5	0.06	8.126	276	**<.001**

Table 3 presents the results of the quantitative comparison of our citizen science data to laboratory data, which revealed no significant differences except in the other’s visual cues and memory versus pointing tasks. Dogs were significantly quicker to approach forbidden food for experimenters in Call et al [14] than citizen scientists reported for their dogs. Dogs tested by citizen scientists were more reliant on their memory in the memory versus pointing task than observed by experimenters in Szetei et al [44].

Qualitative comparisons were conducted where quantitative comparisons were not possible. Whereas the initial publication on dog contagious yawning found that 72.4% of dogs yawned in the experimental but not the control condition [45], we did not see the same effect here. In the combined citizen science dataset there was no significant effect of condition, with 24.9% of dogs yawning in the experimental condition, and 20.8% of dogs yawning in the control condition. We did, however, find a relatively weak effect in the Beta dataset (Table 4). Erdőhegyi et al [46], on which our inferential reasoning task is based, had similar results to ours with the median success for both at or below chance levels (Table 4).

The results from participants who used the redo button did not differ (S2 Table) from participants who did not use this option. The redo button was used too rarely in all but the eye contact and watching conditions to allow for analysis. In eye contact and watching, in which the minimum threshold of participants was reached, there was no difference in results from those who used the redo button and those that did not (eye contact task, df = 25.25, t = -1.04, p = .31; watching, df = 35.68, t = 1.74, p = .09, Welch independent t-test). Finally, the comparison of results from trainers and non-trainers revealed no significant differences in the dogs tested by each group on any of tasks (S3 Table).

**Fig 3:**
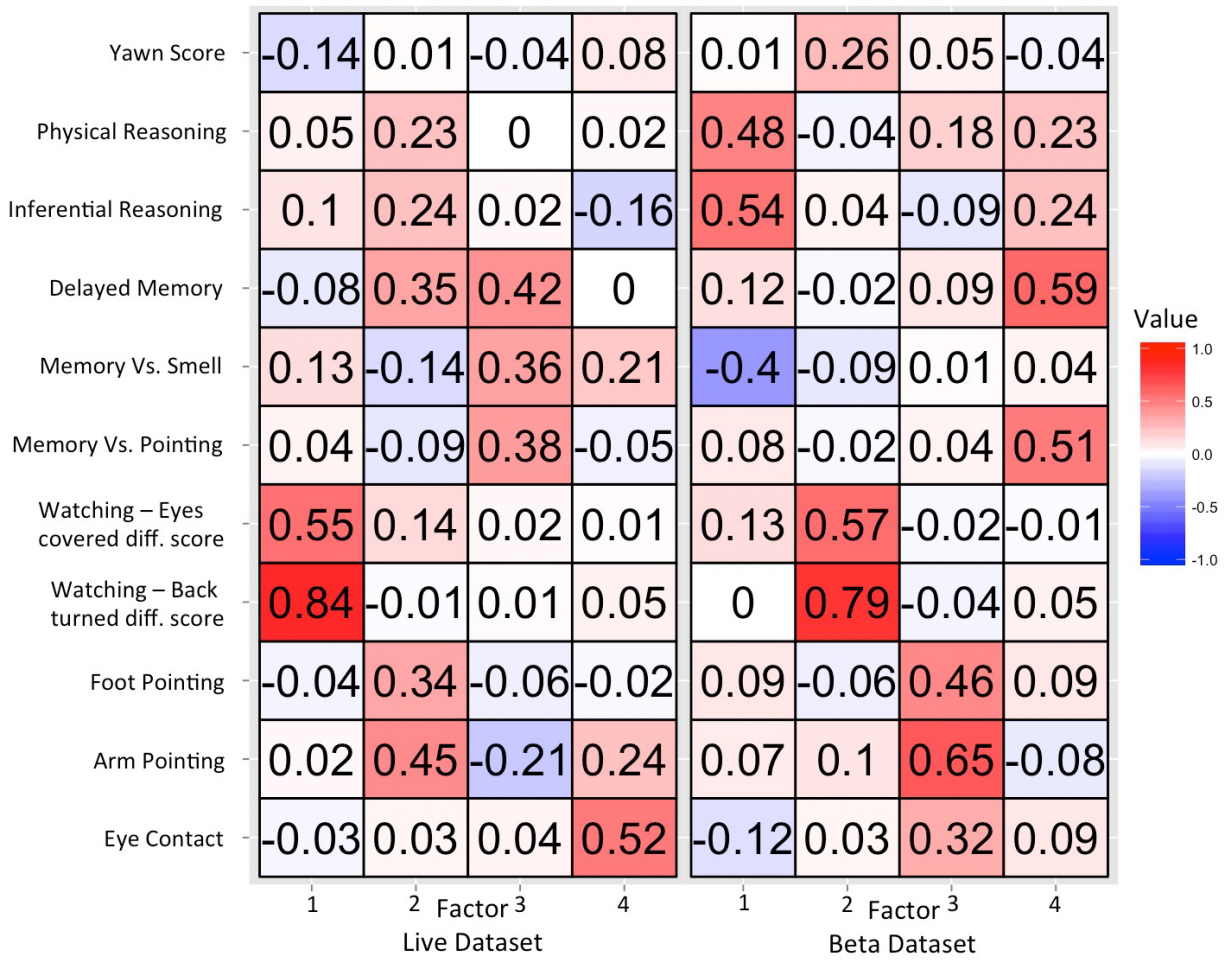
Factor loadings from exploratory factor analyses of the (A) Beta, and (B) Live datasets. Both datasets were best described by four factor models consisting of factors related to gesture comprehension, memory, and cunning, with a fourth factor that varied between the Beta an Live datasets. The order of factors varied between datasets but the following factors resemble one another between analyses (Live PA1 & Beta PA2; Live PA2 & Beta PA3; Live PA3 & Beta PA4. The remaining factors (Live PA4 & Beta PA1) had no clear analog in the other dataset.

Fig 3 presents the results from the factor analysis for the Beta and Live datasets. The factor analysis revealed that a four-factor solution explained 23% and 30% of variance in the Beta and Live datasets, respectively. The factor solution was remarkably consistent between datasets. To interpret the latent variables in the model we assessed the shared characteristics between variables with loadings ≥ .30 on each factor [47]. First, in both datasets the foot pointing and arm pointing tasks load together on the same factor. In the Beta dataset the eye contact exercise also loaded positively on this factor, whereas in the Live dataset the delayed memory task also loaded on this factor. Based on the common loadings from the arm pointing and foot pointing tasks between the live and beta analyses we infer that this factor corresponds to skills for understanding cooperative communication.

Second, in both datasets the difference scores between eyes covered and watching as well as back-turned and watching loaded together on an independent factor. We interpret this factor as reflecting a general tendency for reasoning about others’ visual access, or intelligent disobedience. Third, in both datasets the memory vs. pointing task and the delayed cup task loaded together on the same factor. In the Live dataset, but not the Beta dataset, the memory vs. smell task also loaded highly on this factor. Thus we interpret this factor as corresponding to memory related processes. The remaining factor differed between the beta and live datasets. In the Beta dataset the physical reasoning and inferential reasoning tasks loaded positively on this factor, which was also loaded negatively by the memory vs. smell task. In the Live dataset the eye contact task loaded highly on its own factor, with no other loadings ≥ .30. Thus the factor models for both datasets were consistent with independently covarying skills related to communication, understanding others’ visual perspectives, and memory.

## Discussion

The results of our citizen scientists closely resemble those obtained using conventional laboratory approaches when considering each task individually. Most findings also replicate internally as both the Beta and Live dataset revealed similar patterns even though to be conservative we considered them separately throughout our main analyses. This included the factor analysis that suggested that each dataset was best explained by as many as four cognitive factors with the loading patterns for three factors replicating across the two datasets. Thus, we found initial support that citizen scientists can produce scientifically useful data regarding dog cognition and that multiple cognitive factors best explain individual differences in dog cognition.

Considering each test individually we largely replicate significant effects previously observed in laboratory work in almost every test where a comparison was possible. Dogs used information provided by human gestures when deciding which piece of food to retrieve. They did this even though both choices were visible and they were not differentially rewarded within the test session. They also used the familiar arm gesture and a novel foot pointing gesture at similar levels. Dogs were very successful at locating food they had observed being hidden. They relied on their memory instead of following a deceptive human gesture or the olfactory cues potentially given off by a hidden treat when its location was surreptitiously switched from where the dog had initially seen it hidden. They also were able to locate a treat they had seen hidden after increasing delays of up to two and half minutes. Dogs also solved the solidity task by choosing the paper propped up by the hidden treat significantly more than the paper lying flat that could not have food underneath. Meanwhile, positive but relatively weak evidence was observed for contagious yawning and the effect of the human watching vs. not watching when dogs were prohibited from eating a treat on the ground. Contagious yawning was observed in the Beta but not the Live data set echoing variability in contagious yawning in the published literature. Dogs showed sensitivity to being watched in the Live data set only when their owner covered their eyes but not when they turned their back. They showed no sensitivity to being watched in the Beta data set. Finally, we found no evidence of dogs using the principle of exclusion in locating hidden food during the inferential reasoning task in either the Beta or Live data sets.

These findings largely replicate previous laboratory results that utilized methods closest to what was provided to our citizen scientists (see Table 3). Five of the seven cognitive tasks that allow for quantitative comparison do not reveal significant differences. Arm pointing, foot pointing, memory versus smell, memory delay, and physical reasoning all produce similar findings as observed in published studies. The memory versus pointing exercise is also consistent with the previous finding that dogs do not rely on a pointing gesture when they have just seen a treat being hidden in another location. However, our citizen science data revealed a strong preference by dogs to rely on their memory over a deceptive pointing gesture whereas previously published data found that dogs simply chose at chance levels [44]. Qualitative comparison reveals that the median performance in the inferential reasoning task is 50% in both the citizen science data and Erdőhegyi et al [46]. Although, in our Live data set dogs tested by citizen scientists actually performed significantly below chance preferring to choose the cup they were shown was empty (i.e. potentially misinterpreting the demonstration as communicative similar to Braeuer et al [13]. While we did not see the high level of contagious yawning reported in Joly-Mascheroni et al [45], this may not be surprising since the phenomenon has not been consistently replicated by other labs (e.g. see Romero et al [48] for positive and O’Hara et al [49] for negative results).

The citizen data that differed most from previous published work was the watching exercise. Dogs tested by citizen scientists were reported to wait 10–15 seconds longer in each of the three conditions than in Call et al [14]. Moreover, while dogs’ sensitivity to human eyes and head direction has been observed several times in the published literature, our data did not replicate this seemingly robust finding [14, 16, 18, 50]. Only by considering the Live dataset in isolation is there any evidence that dogs were sensitive to being watched when retrieving forbidden treats. It may be that methodological differences between laboratory techniques and our citizen science approach underlie these differences (see SI). For example, this measure may be more sensitive to who is conducting the experiment than other tasks since here it was always a familiar owner but in conventional studies it is often an unfamiliar experimenter (e.g. [14]). In addition, dogs may behave differently in this context at home relative to an unfamiliar cognitive testing center. Finally, the difference may be due to different approaches to sampling. Some laboratory studies have used selection criteria when choosing subjects that were based on pretests that measure a dog’s level of obedience before accepting them into the test while here no pretest was utilized [18].

By definition citizen science is going to produce a qualitatively different type of data than conventional laboratory approaches [51]. In addition, each discipline will face its own unique challenges implementing citizen science research [28]. Our citizen science program was designed to take in to consideration the challenges that participants might have in conducting behavioral experiments reliably [51]. We only included cognitive exercises that naïve volunteers showed skill at completing during pilot observations based on our instructions and written FAQs. Our live dataset had the added protection of a redo button designed to help identify trials in which participants might have made a methodological error. Use of the redo button was consistent with participants using the button to correct the occasional error but generally feeling that they followed the procedures satisfactorily.

A comparison of dog trainers to non-trainers also provides another type of initial test for experimental bias. If familiarity with dog handling altered results, one would predict that the dogs of professional trainers would differ from the general population. However, we found no difference. Future follow-up tests and questionnaires will allow us to further probe this possibility. Finally, post hoc examinations of the results suggest participants did not intentionally or unintentionally manipulate the data in obvious ways. If owners were consistently exaggerating their results, citizen-tested dogs would be predicted to outperform laboratory-tested dogs across tasks. Instead, citizen-tested dogs performed poorly on the most challenging test (inferential reasoning) while performing almost identically to lab-tested dogs on tasks in which dogs are known to excel (using gestures). They also ignored their owner’s pointing gesture in the memory versus pointing exercise and failed to show evidence of sensitivity to watching. This is not the expected pattern if participants were consistently exaggerating results. Together with the replication of previously published phenomena, internal replications between the Live and Beta datasets, and the similarity between results of those who frequently and infrequently used the redo button, it seems that participants were largely willing and able to follow instructions.

Like many other citizen science projects, our data seems to be of sufficient quality that it will help suggest where hypothesis driven research in more conventional research laboratories can be directed to either confirm or extend findings from citizen science [28]. Our factor analysis represents the first example of how citizen science might be applied to broach questions requiring larger datasets than typical in traditional lab-based approaches to studying dog cognition. Our exploratory factor analysis suggests that multiple domains of cognition best explain individual differences. Performance on all of the tasks did not simply load together on one axis that explained the majority of individual variability. Specifically performance in the two gestures, other’s visual cues, and memory tasks were each associated with one another across individuals. We interpret this as being consistent with (independent) cognitive domains related to cooperative-communication, understanding other’s visual perception, and memory related processes. The Beta dataset even suggests a possible fourth reasoning factor that was not observed in the Live dataset. While future research may show a different set or number of factors, our data strongly suggest that dog cognition will not be easily explained by a single factor. While these citizen science results should be considered preliminary, they clearly point to an exciting area where effort in conventional laboratory settings might be focused. When used in conjunction with more conventional and controlled laboratory approaches, citizen science promises to help push the study of dog psychology toward new frontiers.

## Supporting Information

S1 Dataset Stewart_et_al_data. Accompanying dataset. (CSV)

S1 File Supplemental Materials. (DOCX)

S1 Table Supplemental table 1. The live coding scheme for participants via Dognition’s online interface. The question and potential responses that participants answered after each trial in each task.
(DOCX)

S2 Table Supplemental table 2. The number of participants with either zero or at least one use of the “redo” option in each task. More than twenty participants used the redo button in only two tasks in the Live data. Comparing these two tasks reveals no difference in performance between the two groups.
(DOCX)

S3 Table Supplemental table 3. The mean, degrees of freedom and p-value for each comparison between non-trainer (n = 226) and trainer dogs (n = 51) on each task. # of trials (correct) indicates how many repetitions were conducted and which direction was scored as correct in exercises with within trial choices. Pointing / memory means a correct choice was scored when a dog ate the food the human pointed toward or showed them while they hid it. Means for Back Turned and Eyes Covered represent difference scores between waiting times in each condition subtracted from the Watching condition. All comparisons were conducted using Welch independent t-tests with the exception of the yawning conditions that used Pearson’s Chi-squared with Yates’ continuity correction.
(DOCX)
