# Supplementary material for: Citizen Science as a New Tool in Dog Cognition Research
Source: PLoS One. 2015 Sep 16;10(9):e0135176. doi: 10.1371/journal.pone.0135176 (PMC4574109; doi:10.1371/journal.pone.0135176)
Supplement: S1 File — (DOCX) [file pone.0135176.s002.docx]

# Supplemental Materials

## Participants

Ten dogs were completed during the initial live period but excluded from the Live dataset analysis. Seven of these dogs were excluded for technical issues relating to the operation and storage of data on the Dognition.com servers. Three dogs met an exclusion criteria based on their time to completion (i.e. the data was entered too quickly to be accurate).

Upon registration citizen scientists were required to choose their dogs breed type (Pure, Cross Breed, Popular Hybrid, or Mixed Breed/Other/I Don’t know). If the citizen scientist selected Pure or Popular Hybrid, they were allowed to select a follow-up option from a list based on AKC breeds or hybrids. If the citizen scientist selected Cross Breed, they were given two fields and encouraged to select the two breeds the dog is a mix of. If participants selected Mixed Breed/Other/I Don’t Know then they were asked to select which of those three (Mixed Breed, Other, or I Don’t Know) best described their dog.

## Procedures

### Contagious yawning

For the contagious yawning task the procedure was based on Joly-Mascheroni et al, (2008). Participants watched a video of instructions that included examples of dogs yawning. Participants were instructed to sit on the floor with the dog, but not to touch or interact with the dog at any point during the yawning conditions. In the control condition the participant was prompted to say the word “yellow” every 5 seconds for 30 seconds. In the experimental condition, the participant was instructed to yawn clearly and audibly every 5 seconds for 30 seconds. Once this 30 second period ended, a 2 minute timer started. During the 30-second manipulation and the 2 minutes that followed the participants were to observe if the dog yawned. They were prompted to code whether the dog yawned or not after each trial (they were not asked for a frequency). There was only one trial of each condition and they were not counterbalanced across subjects. All subjects received the control condition first.

### Eye Contact

Procedurally the eye contact task was inspired by Nagasawa et al (2009).

Participants were first instructed to conduct a warm-up. In the warm-up condition, the participant was instructed to stand facing their dog with the dog standing or sitting ~1.8 meters away. The participant was then prompted to call the dogs’ name, show the dog a small treat, and place that treat directly beneath their eye and to start the 10 second count down timer. Once time elapsed, the participant was prompted to give the dog the treat. No coding was required and the warm-up was repeated three times.

The experimental condition was the same as the warm-up with the exception that the participant was instructed to stop the timer once the dog broke eye contact for more than two seconds. If the dog never broke eye contact the timer ended at 90 seconds. Three trials of this condition were conducted.

### Arm and Foot Pointing

The arm pointing task was based on Gácsi et al (2009) while the foot pointing task was based on Lakatos et al (2009). In the pointing warm-up the dog was introduced to the two potential locations to find a treat. The participant was instructed to call the dog’s name, show the dog a treat, and place the treat down on the ground at arm’s length to either the left or right. The instructions indicated which side to place to food on in each trial. Counterbalancing was between subjects, and within subjects, and participants never gestured to the same side on two consecutive trials. Once the participant had placed the treat, they were instructed to release the dog while giving a release command. After each trial the participant was asked to record if the dog retrieved the treat. There were 6 trials in this condition.

The two experimental conditions were identical to the warm-up with the exception that two identical food treats were placed at arms length on either side. Participants were instructed which food piece they were to gesture toward and to allow the dog to retrieve both food pieces while recording which food piece the dog approached first. A first approach was illustrated in the video and was defined as crossing the plane created by the post-it notes on the left or right. In the arm pointing condition participants were to point by extending their arm, hand and index finger toward the dog while standing equidistant between the two pieces of food. In the foot pointing condition participants were instructed to take one step toward one piece of food making them physically closer to one piece of food while extending their leg and food in the direct of the other piece of food. In both experimental conditions gaze and gesture was to be directed at the piece of food being indicated until the dog made their choice. Six trials were administered in each condition

### Other’s Visual Cues

The other’s visual cues task was procedurally based on Call et al (2003). In the baseline watching condition the dog was positioned 1.8 meters from the participant. The participant was then instructed to say the dog’s name and “No” or “Leave it” twice, clearly and loudly, while placing a treat in front of them on the ground. Once the participant stood back up, they activated the countdown timer, which they only stopped when the dog approached and ate the forbidden food. The timer automatically stopped after 90 seconds if the dog never approached. The two experimental conditions were identical to the baseline condition except the participant did not watch the treat. In the back turned condition the participant immediately turned their back to the dog while in the eyes covered condition the participant covered his/her eyes with their hands. If a partner was helping then they were also instructed to turn their back. Participants were instructed to look over their shoulder every few seconds or to peer through their fingers subtly to detect when the dog approached. Two trials of the back turned and eyes covered condition were conducted. One watching trial was conducted before and after the four experimental conditions. The two back turned conditions always preceded the eyes covered trials.

### Memory Tasks

Dogs were first introduced to finding food hidden underneath cups. The participant started by placing one opaque cup face down on the left or right at arm’s length. In the first condition of the warm-up task, the participant was instructed to call the dog’s name and show the dog a treat. The participant then placed this treat underneath the cup as their dog watched. The second warm-up condition was the same as the one-cup warm-up except the participant placed a cup at arm’s length on both the left and right simultaneously. Then they placed a food treat under one of the two cups as their dog watched. Counterbalancing was automated so that between subjects half of subjects received their first trial on the left or right, within a test subjects received equal number of trials on the left and right, and treats were never hidden on the same side more than two consecutive trials. Once the treat had been placed under the cup, the participant gave a release command. A first approach was illustrated in the video and was defined as crossing between the plane created by the center-left or center right post-it notes. If the dog chose the side with the treat, the dog was allowed to eat the treat. If the dog chose the side without the treat, the participant was instructed to show the dog where the treat was located, but to prevent the dog from eating the treat. After each trial the participant was prompted to record if the dog retrieved the treat. Four trials of each warm-up condition were administered for a total of eight warm-up trials.

The memory versus smell condition was based on a similar comparison in Szetei et al (2003). This task was identical to the two-cup warm-up. However, while the dog’s view was occluded the treat was switched to the opposite cup in which it was initially hidden (suggestions for blocking the dog’s view included (1) covering the dogs eyes with the partners hands, (2) turning the dog around while distracting it, or (3) kneeling in front of the dog while holding its head). Four trials were administered. The memory versus pointing condition was also was based on a similar condition in Szetei et al (2003). Memory versus pointing was identical to the two-cup warm-up with the exception that once the food was hidden the participant extended their arm, hand and index finger toward the empty cup. Six trials of this condition were conducted. Finally, the delayed cup procedure was based on Fiset et al (2003) and MacLean et al. (unpublished data). The delayed cup task was the same as the two-cup warm-up except that after the food was placed participants waited increasingly longer delays before releasing their dog to search for the hidden food. In the first trial the countdown timer ran for 1 minute, increasing by 30 seconds per trial for 3 more trials. The delay for the last trial lasted for 2 minutes and 30 seconds.

### Reasoning Tasks

The inferential reasoning task was based on the procedure of Erdőhegyi et al (2007). In the inferential reasoning warm-up the participant was instructed to place two cups at arm’s length to the left and right. Both were placed on their side with the inside of the cup facing the dog. The participant was then instructed to call the dog’s name and show the dog a treat. The participant then blocked the dog’s view of the treat in their hand with an opaque occluder. They then baited one cup while sham baiting the other. Regardless of where the treat was hidden the participant was instructed to always move from the right cup to the left cup. Counterbalancing was automated so that between subjects half of subjects received their first trial on the left or right, within a test subjects received equal number of trials on the left and right, and treats were never hidden on the same side more than two consecutive trials.

Once the participant had visited both the right and left cup, they placed the barrier behind them and left the cups laying flat with open sides facing the dog (so that the dog could easily see the treat in one of the cups). To help the dog see the treat clearly the participant lifted both cups simultaneously, bringing them together at the dog’s approximate eye level. They then placed them upside down to hide the food’s location. Dogs simply had to remember where they saw the food hidden when released. After each trial the participant was asked to record if the dog retrieved the treat. Six trials of this warm-up were conducted.

In the experimental condition of the inferential reasoning task the procedure is the same as the warm-up except after baiting the cups they were flip upside down without showing the dog where the food was hidden. Then the participant showed the dog which cup was empty by lifting the empty cup and bringing it to the dog’s approximate eye level. After placing the empty cup back down, the dog is released to search. Four trials of this experimental condition were conducted.

In the physical reasoning warm-up the participant folds two pieces of standard U.S. (8.5’X11’) paper width-wise so that when placed on the ground, they remain flat. One piece of paper was then placed at arm’s length on the left or right. The participant was then instructed to call the dog’s name and show the dog a treat. They then occluded the baiting of the hiding location by holding an opaque barrier in front of the hand with food. The participant then wedged the treat in the crease of the paper, propping one side of the paper up. Once the participant had hidden the treat, they placed the barrier behind them and the dog was released to search. If the dog approached close enough to touch the paper, the participant could either let the dog open the paper or fold the paper down to help the dog access the food. If the dog did not approach the paper after 90 seconds, the participant would show the location of the treat to the dog and allow him/her to eat it. After each trial the participant was asked to record if the dog retrieved the treat. There were 4 trials in this condition.

The physical reasoning condition was based on Bräuer et al, (2006). In the physical reasoning experimental condition the procedure was the same except the participant placed a folded piece of paper on the left and right at arm’s length. They then occluded the baiting as before as they propped one of the pieces of paper up using the treat. Counterbalancing was automated so that the first side baited alternated equally between left and right across dogs and within the test session of any one dog. After each trial the participant was asked to record if the dog retrieved the treat. There were 4 trials in this condition.

## Comparison to previously published findings

Methodologically every task presented to our participants was modified from the published study on which they were based. Most changes were to simplify procedures or reduce the total number of trials needed. For example, in the arm and foot pointing exercises food was placed as the dog watched while the experimenter pointed to one while both pieces of food were visible to the dog. This differs from most previous studies with dogs including Gácsi et al (2009) and Lakatos et al (2009) in which one piece of food is hidden in one of two locations. This change was based on MacLean & Hare (2014) where hiding or leaving food visible had no effect on the performance of apes in using human pointing gestures. Leaving food visible for dogs during piloting significantly reduced the number of warm-up trials necessary for this task as well as the procedural mistakes made by the dog owners. Each experiment differed in important ways from the original on which it was based: the time to observe a yawn was shorter, food was placed near the participant’s face in the eye contact exercise, the owner covered their eyes with their hands instead of just closing them in the visual cues task, only two hiding locations were used in delayed memory instead of four (similar to MacLean et al unpublished but unlike Fiset et al., 2003), and a forced choice was used in the warm-up of the physical reasoning task to name a few. Given all the methodological changes, what is remarkable then is how similar the current findings are to the published procedures that differ procedurally. Moreover, we can assume citizen scientists are far less methodologically consistent than trained experimentalists. This suggests that the previous findings on which Dognition.com tasks are based are relatively robust to methodological noise. The one obvious exception seems to be the discordant findings in the other’s visual cues task. It may be that covering of eyes with hands, being tested at home by an owner, or having a second experiment look over their shoulder significantly altered the performance of citizen dogs (note: Looking over the shoulder was done during the back turned condition because the experimenter could not see if the dog was approaching the forbidden food and prevent the dog from taking it fast enough. This would have meant many dogs were rewarded for quickly going to obtain food when the experimenter could not see them, but not while the experimenter was watching. To maintain non-differential rewarding the second experimenter stood behind the dog, looked over their shoulder briefly and warned of when the dog first began approaching the forbidden food).

Welch independent t-test were used for all quantitative comparisons unless otherwise stated. The arm-pointing condition was compared to the “distal momentary pointing task” of Gácsi et al. (2009). The foot-pointing condition was compared to “pointing with leg” from experiment two in Lakatos et al. (2009). The latency scores of the citizen science dataset in each condition were compared to the latency scores in the watching, back turned, and eyes covered conditions of Call et al. (2003). Although the maximum trial length in Call et al. (2003) was 180 seconds, no dogs had higher latencies than 90 seconds – permitting quantitative comparison. Memory versus pointing was compared to “deceptive pointing contra hiding” and memory versus smell was compared to “deceptive hiding” from Szetei et al (2003). Due to the sample size use in Szetei et al (2003) a non-parametric Wilcoxon test was used for comparison. The delayed memory task was compared to unpublished data, gathered by the Duke Canine Cognition Center during 2011 (n=49 dogs) using a 2-sample test for equality of proportions with continuity correction. The dogs were enrolled in an assistance dog program and were being tested as part of a larger battery of tests. Procedurally the delayed memory test used with the assistance dogs was almost identical to that used by Dognition.com with the exception that 1) each assistance dog was given 4 trials each with a 30 second and 60 second delay 2) the hiding locations were buckets not cups. For our comparison we only used the first trial of the sixty second condition from the assistance dogs since citizen dogs only received one trial at 60 seconds. The physical reasoning task was compared to the “Causal: Visual Cues-Shape” condition of Bräuer et al. 2006. The main notable difference between the two protocols was the material that was propped up by the food. In Bräuer er al. (2006) the material was a rigid wooden board, where as in the citizen science dataset the material was a folded piece paper.

For the qualitative comparison of the inferential reasoning task we used experiment one- ‘empty only’ condition of Erdőhegyi et al. (2007). Although mean success is not reported, Erdőhegyi et al. (2007) do report the median success of the sample, which we compared to the citizen data. Contagious yawning data was qualitatively compared to Joly-Mascheroni et al. 2008 since it most closely matched the Dognition.com methodology. In an experimental condition an experimenter continuously yawned for 5 minutes while in a control condition the same experimenter displayed “non-yawning mouth-opening actions”. The main differences between the Dognition.com protocol and this initial task include 1) the shorter trial length for Dognition.com and 2) the use of familiar owners for Dognition.com compared to unfamiliar experimenters – although familiarity should only increase the rate of yawning overall (Romero et al, 2013).

# Supplemental References

Joly-Mascheroni, R. M., Senju, A., & Shepherd, A. J. (2008). Dogs catch human yawns. Biology letters, 4(5), 446-448.

Nagasawa, M., Kikusui, T., Onaka, T., & Ohta, M. (2009). Dog's gaze at its owner increases owner's urinary oxytocin during social interaction. Hormones and behavior, 55(3), 434-441.

Szetei, V., Miklósi, Á., Topál, J., & Csányi, V. (2003). When dogs seem to lose their nose: an investigation on the use of visual and olfactory cues in communicative context between dog and owner. Applied Animal Behaviour Science, 83(2), 141-152.

Fiset, S., Beaulieu, C., & Landry, F. (2003). Duration of dogs' (Canis familiaris) working memory in search for disappearing objects. Animal Cognition, 6(1), 1-10.

MacLean, E., & Hare, B. (2014).Bonobos and chimpanzees exploit helpful but not prohibitive gestures. *Behaviour.*
