# Supplementary material for: Citizen Science as a New Tool in Dog Cognition Research
Source: PLoS One. 2015 Sep 16;10(9):e0135176. doi: 10.1371/journal.pone.0135176 (PMC4574109; doi:10.1371/journal.pone.0135176)
Supplement: S1 Table — The live coding scheme for participants via Dognition’s online interface. The question and potential responses that participants answered after each trial in each task. (DOCX) [file pone.0135176.s003.docx]

**Supplemental Table 1:** The live coding scheme for participants via Dognition’s online interface. The question and potential responses that participants answered after each trial in each task.

| **Task** | **Question** | **Responses** |
| --- | --- | --- |
| Yawn Control | Did {dog_name} yawn? | No,Yes |
| Yawn Experimental | Did your dog yawn? | No,Yes |
| Eye Contact Warm-up | NA | Next |
| Eye Contact | NA | Stop timer |
| Pointing Warm-up | Did {dog_name} chose the side with the treat? | No,Yes |
| Arm Pointing | Did {dog_name} chose the side you were pointing at? | No,Yes |
| Foot Pointing | Did {dog_name} chose the side you were pointing at? | No,Yes |
| Watching | NA | Stop timer |
| Back Turned | NA | Stop timer |
| Eyes Covered | NA | Stop timer |
| One Cup Practice | Did {dog_name} retrieve the treat? | No,Yes |
| Two Cup Practice | Did {dog_name} retrieve the treat? | No,Yes |
| Memory vs. Pointing | Did {dog_name} retrieve the treat? | No,Yes |
| Memory vs. Smell | Did {dog_name} retrieve the treat? | No,Yes |
| Delayed Memory | Did {dog_name} retrieve the treat? | No,Yes |
| Inference warm-up | Did {dog_name} retrieve the treat? | No,Yes |
| Inferential Reasoning | Did {dog_name} retrieve the treat? | No,Yes |
| Physical warm-up | Did {dog_name} retrieve the treat? | No,Yes |
| Physical Inference | Did {dog_name} retrieve the treat? | No,Yes |
