# Supplementary material for: Citizen Science as a New Tool in Dog Cognition Research
Source: PLoS One. 2015 Sep 16;10(9):e0135176. doi: 10.1371/journal.pone.0135176 (PMC4574109; doi:10.1371/journal.pone.0135176)
Supplement: S2 Table — The number of participants with either zero or at least one use of the “redo” option in each task. More than twenty participants used the redo button in only two tasks in the Live data. Comparing these two tasks reveals no difference in performance between the two groups. (DOCX) [file pone.0135176.s004.docx]

**Supplemental Table 2:** The number of participants with either zero or at least one use of the “redo” option in each task. More than twenty participants used the redo button in only two tasks in the Live data. Comparing these two tasks reveals no difference in performance between the two groups.

| **Exercise** | **>0 redo** | **0 redos** | **Mean >0 redo** | **Mean 0 redos** | **T** | **P** | **df** |
| --- | --- | --- | --- | --- | --- | --- | --- |
| Yawn Control | 13 | 264 | .2307 | .2348 |  |  |  |
| Yawn Experimental | 10 | 267 | .10000 | .2359 |  |  |  |
| Eye Contact | 22 | 255 | 41.6s | 46.796s | -1.04 | .308 | 25.255 |
| Arm Pointing | 4 | 273 | 2.75 | 4.01 |  |  |  |
| Foot Pointing | 2 | 275 | 2.5 | 3.98 |  |  |  |
| Watching | 29 | 248 | 61.24s | 49.70s | 1.73 | .091 | 35.681 |
| Back Turned | 16 | 261 | 56.84s | 51.43s |  |  |  |
| Eyes Covered | 8 | 269 | 66.68s | 51.30s |  |  |  |
| Memory vs Pointing | 2 | 275 | 2.00 | 3.81 |  |  |  |
| Memory vs Smell | 6 | 271 | 3 | 2.94 |  |  |  |
| Delayed Memory | 8 | 269 | 3.37 | 3.1 |  |  |  |
| Inferential Reasoning | 1 | 276 | 1 | 1.83 |  |  |  |
| Physical Reasoning | 5 | 272 | 2.6 | 2.49 |  |  |  |
