# Supplementary material for: Citizen Science as a New Tool in Dog Cognition Research
Source: PLoS One. 2015 Sep 16;10(9):e0135176. doi: 10.1371/journal.pone.0135176 (PMC4574109; doi:10.1371/journal.pone.0135176)
Supplement: S3 Table — The mean, degrees of freedom and p-value for each comparison between non-trainer (n = 226) and trainer dogs (n = 51) on each task. # of trials (correct) indicates how many repetitions were conducted and which direction was scored as correct in exercises with within trial choices. Pointing / memory means a correct choice was scored when a dog ate the food the human pointed toward or showed them while they hid it. Means for Back Turned and Eyes Covered represent difference scores between waiting times in each condition subtracted from the Watching condition. All comparisons were conducted using Welch independent t-tests with the exception of the yawning conditions that used Pearson’s Chi-squared with Yates’ continuity correction. (DOCX) [file pone.0135176.s005.docx]

**Supplemental Table 3:** The mean, degrees of freedom and p-value for each comparison between non-trainer (n=226) and trainer dogs (n=51) on each task. # of trials (correct) indicates how many repetitions were conducted and which direction was scored as correct in exercises with within trial choices. Pointing / memory means a correct choice was scored when a dog ate the food the human pointed toward or showed them while they hid it. Means for Back Turned and Eyes Covered represent difference scores between waiting times in each condition subtracted from the Watching condition. All comparisons were conducted using Welch independent t-tests with the exception of the yawning conditions that used Pearson's Chi-squared with Yates' continuity correction.

|  |  | **Non-trainer** | | **Trainer** | |  |  |  |
| --- | --- | --- | --- | --- | --- | --- | --- | --- |
| **Exercise** | Trial # (correct) | **Mean** | **SE** | **Mean** | **SE** | **T** | **d.f.** | **P** |
| Yawn control | 1 | .23 | .028 | .25 | .061 |  | 1 | X^2^=0.037  p=.84 |
| Yawn experimental | 1 | .24 | .028 | .17 | .053 |  | 1 | X^2^=0.70  p=.40 |
| Eye Contact | 3 | 46.86 | 1.55 | 44.23 | 3.41 | .701 | 73.2 | p=.48 |
| Arm Pointing | 6 (pointing) | 4.0 | .078 | 3.86 | .189 | .774 | 68.3 | p=.44 |
| Foot Pointing | 6 (pointing) | 3.84 | .081 | 4.07 | .152 | -1.37 | 81.2 | p=.17 |
| Back Turned | 2 vs. 2 | -.325 | 1.36 | -3.07 | 2.32 | 1.02 | 88.2 | p=.31 |
| Eyes Covered | 2 vs. 2 | 3.56 | 1.37 | 1.41 | 2.31 | .801 | 89.1 | p=.42 |
| Memory vs. Pointing | 6 (memory) | 3.86 | .131 | 3.5 | .284 | 1.14 | 72.9 | p=.25 |
| Memory vs. Smell | 4 (memory) | 2.96 | .076 | 2.88 | .155 | .450 | 76.2 | p=.65 |
| Delay | 4 (memory) | 3.12 | .069 | 3.19 | .131 | -.486 | 80.0 | p=.62 |
| Inferential Reasoning | 4 (baited cup) | 1.88 | .071 | 1.80 | .125 | .562 | 85.4 | p=.57 |
| Physical Reasoning | 4 (baited occluder) | 2.46 | .069 | 2.60 | .125 | -.969 | 83.1 | p=.33 |
